# Secular Press and Medical Advertisements

**Published:** 1874-08

**Authors:** 


We have received from the State of Alabama a copy of a weekly *newspaper* containing an essay upon yellow fever read before a local medical society. In another column we find a lengthy, leaded and displayed *advertisement*, signed by the author of the essay, proposing to *sell to his medical brethren his secret remedy!* "My plan is, at present, to furnish printed directions with the physic, which directions must be carried out to the letter, or no guarantee. * * * I am old and in feeble health, and I wish to make something out of it before I give the discovery to the world."

We know nothing of the essayist and advertiser. He may be, we doubt not he is, an excellent old gentleman, who has done much good service in his day in the ranks of the profession. We do know that the society, before which the essay purports to have been read, includes many most excellent and high-toned medical gentlemen. We certainly are disposed to entertain a large and liberal charity for the infirm veteran; and yet we can see no reason, in a land where the recognized channels of medical communication with the profession are so abundant as in this, why an essay, read before a medical society, should find its way before the public in the columns of a secular newspaper.

It is sad indeed to know that an aged veteran, worn out in the medical service of the public, should be compelled in his declining years to lift his trembling, withered hand and rend the ethical veil which had so long separated him from dishonor, that he might put meal in the tub. Surely there must be hundreds of true men in his own good State of Alabama, who would cheerfully share their scanty crust with the old soldier rather than see him starved into the sacrifice of his professional self-respect.

